# PATIENT AGE AND SEX DO NOT APPEAR TO INFLUENCE DECISION MAKING AROUND BIOMARKER TESTING FOR COGNITIVE IMPAIRMENT

**DOI:** 10.1093/geroni/igad104.3792

**Published:** 2023-12-21

**Authors:** Mark Monane, Kim Johnson, Joy Snider, R Scott Turner, Jonathan Drake, Julia Ortega, Yan Jiang, Joel Braunstein

**Affiliations:** C2. N DIagnostics, St. Louis, Missouri, United States; Duke University, Durham, North Carolina, United States; Washington University at St Louis, St. Louis, Missouri, United States; Georgetown University, Washington, District of Columbia, United States; The Warren Alpert Medical School of Brown University, Providence, Rhode Island, United States; C2. N Diagnostics, St. Louis, Missouri, United States; Stat4. ward, Pittsburgh, PA, Wexford, Pennsylvania, United States; C2. N Diagnostics, St. Louis, Missouri, United States

## Abstract

The QUIP I Study (ClinicalTrials.gov Identifier: NCT05477056) was a prospective, single-arm study which included 347 older persons (average age 74, 56% women) presenting with signs and symptoms of cognitive impairment. In a subgroup analysis, we measured the effect of a person’s age and sex on clinical decision making around the PrecivityAD® blood biomarker (BBM) test result. The test result was reported as the Amyloid Probability Score (APS), which measures the likelihood of a positive result on an amyloid PET scan. Clinical decision making was recorded by clinician survey pre- and post-BBM testing. Clinician-reported probability of Alzheimer’s disease (“AD”) changed pre-test to post-test from 58% to 23% (Low APS group) and from 71% to 89% (High APS group) (p < 0.0001 for all APS groups). The relationships between APS and change in diagnostic certainty were not significantly different as analyzed by age (p=0.344 for Low APS, p=0.292 for High APS) or sex (p=0.167 for Low APS, p=0.213 for High APS). Overall use of AD drug therapy decreased from 48% to 26% (Low APS group) and increased from 56% to 88% (High APS group) (p < 0.0001 for all APS groups). The relationships between APS and change in medication prescribing were not significantly different as analyzed by age (p=0.4534 for Low APS, p=0.9939 for High APS) or sex (p=1 for Low APS, p=0.931 for High APS). We believe that the current study results help to underscore the usefulness and generalizability of this BBM among older adults in clinical care pathways.

